# Spatial disease dynamics of free-living pathogens under pathogen predation

**DOI:** 10.1038/s41598-017-07983-2

**Published:** 2017-08-10

**Authors:** Tommi Mononen, Lasse Ruokolainen

**Affiliations:** University of Helsinki, Department of Biosciences, Helsinki, FI-00014 Finland

## Abstract

The epidemiological dynamics of potentially free-living pathogens are often studied with respect to a specific pathogen species (e.g., cholera) and most studies concentrate only on host-pathogen interactions. Here we show that metacommunity-level interactions can alter conventional spatial disease dynamics. We introduce a pathogen eating consumer species and investigate a deterministic epidemiological model of two habitat patches, where both patches can be occupied by hosts, pathogens, and consumers of free-living pathogens. An isolated habitat patch shows periodic disease outbreaks in the host population, arising from cyclic consumer-pathogen dynamics. On the other hand, consumer dispersal between the patches generate asymmetric disease prevalence, such that the host population in one patch stays disease-free, while disease outbreaks occur in the other patch. Such asymmetry can also arise with host dispersal, where infected hosts carry pathogens to the other patch. This indirect movement of pathogens causes also a counter-intuitive effect: decreasing morbidity in a focal patch under increasing pathogen immigration. Our results underline that community-level interactions influence disease dynamics and consistent spatial asymmetry can arise also in spatially homogeneous systems.

## Introduction

While epidemiological theory tends to concentrate on obligate pathogens transmitted from host to host (such as measles), many pathogens are actually opportunistically infecting hosts from the environment. Environmental opportunistic pathogens (EOPs) are free-living; they can survive and reproduce in the environment without the presence of a host species (e.g., Vibrio cholerae environmental reservoirs found in Haiti^1^), but can invade host individuals under favorable conditions. As the long-term survival of the pathogens is independent of hosts, they can cause highly lethal diseases and wipe out even entire host populations^2, 3^. For saprotrophic organisms, killing a host can promote pathogen transmission by an increased growth rate in the dead body^4^. This kind of behavior is highly unlikely for obligate pathogens as the death of an entire host population would also eventually eradicate the pathogens. Examples of EOP diseases include cholera, tetanus, tuberculosis, legionnaires’ disease and listeria in humans^5^, anthrax in livestock^6^, Columnaris disease in fish^2^, and white nose syndrome in bats^7^. These diseases are causing deaths as well as economic losses with respect to production animals^8^.

Environmental pathogens living in soil or water encounter predation and competition for resources, which both are decreasing pathogen densities in nature. For example, protozoan predators have been shown to play a significant role in regulating V. cholerae in coastal marine waters^9^. These bactiovorous predators include protozoa (ciliates, fagellates and amoebae) as well as more complex organisms (e.g. nematodes)^10^. There also exist predatory bacteria, which are using other bacteria as an energy source^11^. When the density of EOPs is low, they are less likely to infect hosts, while when densities become higher, infections start to emerge in the host population^12^. At the same time, predation of the pathogens can increase due to increased consumer densities^13^. However, this predation cannot eradicate the whole pathogen population^14, 15^, which enables also future outbreaks.

Earlier theoretical studies on environmental pathogens have mainly concentrated on local dynamics in a single habitat patch. These studies have demonstrated how outside-host competition can affect disease outbreaks and pathogen invasion ability^16, 17^, and how the predation of pathogens^8^ or their vectors^18^ change disease dynamics. Environmental fluctuations have also been shown to play a role in pathogen outbreaks^19, 20^. In general, temporal effects in a single habitat patch are not enough to describe more complex phenomena taking place in nature. An essential part of real-world population dynamics comes from individuals’ movements between habitats.

Dispersal is an important factor contributing to the spread and persistance of disease epidemics^21, 22^. Obligate pathogens infecting a single isolated host population will run out of susceptible hosts due to acquired host population immunity, or local host extinction. On the other hand, pathogens that can disperse effectively can more easily find the new populations of susceptible hosts. Even if one host population becomes immune, epidemics in other localities, as well as evolutionary changes (or random mutations), can allow for future epidemics in the focal population (e.g. influenza). In real life, diseases spread in spatially heterogeneous environments, and therefore disease dynamics cannot be modeled accurately without using spatial information, influencing spread, emergence, severity^23^, frequency^24^ and persistance^25^ of an epidemic.

While environmental opportunistic pathogens (EOPs) can survive without hosts, their disease dynamics can also be strongly affected by spatial processes. Existing spatio-temporal studies usually predict the spread of epidemics caused by a single pathogenic agent based on host movement^26, 27^. For example Mari *et al*. noticed that including a human mobility assumption leads to greatly improved prediction of the spread of cholera epidemics^28^. In this paper, we investigate the spatio-temporal metacommunity dynamics of an environmental opportunistic pathogen in a simple two-patch system, where pathogens are subject to predation in both habitat patches. Each patch contains a population of susceptible and infected hosts, pathogens, and consumers. We consider different scenarios, where either pathogens, consumers or hosts are able to move between patches via random dispersal. Our main hypothesis is that community structure coupled with dispersal will change conventional disease dynamics.

## Model and Methodology

Our deterministic continuous-time model consists of two parts: an SIS model for a host-pathogen interaction, coupled with a predator-prey model to describe the interaction between consumers and pathogens. We assume that hosts, pathogens, and consumers occupy two habitat patches, which are connected via dispersal. For host dynamics we assume carrying capacity limited growth, the susceptible-infective-susceptible cycle, and disease mortality. Pathogen dynamics take into account pathogen growth, density-dependent mortality, shedding from infected hosts (hence, pathogen benefits from causing infections), and loss to predation. In consumer dynamics we model, pathogen population size related growth and consumer population decline in the absence of the pathogen. We assume that dispersal rates for susceptible and infected hosts are equal (unless otherwise stated) and we also include dispersal rates for consumers and pathogens.

### Epidemiological part

The model consists of two habitat patches denoted by subindices *i* and *j*. The epidemiological part (SIS model) is modeled by two equations (per patch):

1 $$\frac{d{S}_{i}}{dt}={r}_{{\rm{host}}}({S}_{i}+{I}_{i})-{r}_{{\rm{host}}}{S}_{i}(\frac{{S}_{i}+{I}_{i}}{{K}_{{\rm{host}}}})-\beta {S}_{i}f({P}_{i})+\delta {I}_{i}+{d}_{{\rm{host}}}({S}_{j}-{S}_{i}),$$

2 $$\frac{d{I}_{i}}{dt}=\beta {S}_{i}f({P}_{i})-{r}_{{\rm{host}}}{I}_{i}(\frac{{S}_{i}+{I}_{i}}{{K}_{{\rm{host}}}})-\nu {I}_{i}-\delta {I}_{i}+{d}_{{\rm{host}}}({I}_{j}-{I}_{i}),$$

where *S*_*i*_ and *I*_*i*_ are the populations sizes of susceptible and infected hosts in patch *i*. The pathogen population size *P*_*i*_ is discussed later along with the consumer-pathogen dynamics (see below). Interpretation of all parameters is explained in Table 1. In the susceptible equation, the first two terms are for host growth and carrying capacity related mortality (a logistic growth function). The third term gives pathogen abundance related infection rate and the fourth term gives the rate of host recovery. In the equation for infected, the third term gives a mortality rate for infected hosts. The last term in both differential equations is a host dispersal term, indicating that hosts disperse at a constant rate *d*_host_ between the patches *i* and *j*. The infection rate *βS*_*i*_ *f*(*P*_*i*_) depends on a sigmoidal infectivity response^12, 16^:

3 $$f({P}_{i})=\frac{{({P}_{i}/{{\rm{ID}}}_{50})}^{\kappa }}{1+{({P}_{i}/{{\rm{ID}}}_{50})}^{\kappa }},$$

where ID_50_ is a half saturation constant that indicates the dose at which 50% of hosts are infected. The parameter *κ* defines the steepness of the sigmoidal infectivity response (see Fig. S5a). This response describes natural infectivity phenomenon, where (I) a dose has to be larger than some threshold to cause infections and when (II) the number of infected hosts gets close to full saturation, the growth rate of new infections decreases. Such a response can arise, e.g., when the host immune system can neutralize a small number of invaders, and the infection process (onset of disease) is not instantaneous^12^.

**Table 1:** A table of model parameters, their explanations and parameter values that are used in the simulations.

Parameter	Interpretation	Values used in simulations
**Epidemiological part**		
*r*_host_	Host growth rate (per capita)	0.01
*K*_host_	Host carrying capacity	100, 200 (default 100)
*β*	Maximum infectivity	5
*δ*	Host recovery rate from infection	0.7
*ν*	Infection kill rate	0.001–0.3 (default 0.01)
*d*_host_	Host dispersal rate	0–1.0
**Consumer-pathogen part**		
*r*_path_	Pathogen growth rate (per capita)	1.3
*μ*	Pathogen mortality (per capita)	0.007
*λ*	Additional in-host pathogen production (shedding)	0.5
*b*	Consumer growth rate/consumption rate	0.35
*c*	Consumer population decline (per capita)	0.36
*d*_path_	Pathogen dispersal rate	0–0.1 (default 0.0)
*d*_con_	Consumer dispersal rate	0–1.0
**Sigmoidal infectivity response**		
*κ*	Slope parameter of the sigmoidal infectivity function	1, 2, 3, 4, 5 (default 3)
ID_50_	Infectious dose at which 50% of hosts are infected	200
**Holling’s functional response**		
*a*	Attack rate	0.025–1.125 (default 0.55)
*h*	Handling time	0.025–1.125 (default 0.45)
*q*	Shape parameter of the response	1.0–2.0 (default 1.0)

### Consumer-pathogen part

The consumer-pathogen interaction is modeled with:

4 $$\frac{d{P}_{i}}{dt}={r}_{{\rm{path}}}{P}_{i}-\mu {P}_{i}^{2}+\lambda {I}_{i}-bg({P}_{i}){C}_{i}+{d}_{{\rm{path}}}({P}_{j}-{P}_{i}),$$

5 $$\frac{d{C}_{i}}{dt}=bg({P}_{i}){C}_{i}-c{C}_{i}+{d}_{{\rm{con}}}({C}_{j}-{C}_{i}),$$

where *C*_*i*_ is the size of consumer population in habitat patch *i*. In the pathogen equation, the first two terms are for pathogen growth and density depended mortality (a logistic growth function). The important third term is *λI*_*i*_, which is a continuous shedding of pathogens from infected hosts, indicating that pathogens can reproduce more effectively inside infected host individuals. The fourth term determines a pathogen consumption, where parameter *b* determines a pathogen consumption rate. In the consumer equation, the second term give the intrinsic decay rate for the consumer population. The last terms describe pathogen and consumer movements between patches. The relationship between predation and pathogen population size is modelled non-linearly using Holling’s functional response:

6 $$g({P}_{i})=\frac{a{({P}_{i})}^{q}}{1+h\cdot a{({P}_{i})}^{q}},$$

where *h* is handling time of pathogens and *a* is a constant defining attack rate. When *q* = 1, the response is of type II, and when *q* > 1 the response approaches a sigmoidal type III -function^29^. Here we assume *q* = 1, if not stated otherwise. The handling time tunes the overall aggressiveness of the consumers; with a short handling time, more pathogens can be eaten per time unit. The attack rate determines effectively how strongly consumers attack a pathogen population at near-zero density (see Fig. S5b). If handling time is short, aggressive consumers make dynamics fast and rough by rapidly consuming the entire pathogen population. On the other hand, ineffective consumers (long handling time) let a pathogen population to survive for a long time. Therefore the handling time affects to conversion efficiency^30^: with a short handling time, energy moves efficiently, and with a long handling time inefficiently, from the trophic level of pathogens to the trophic level of consumers. With a high attack rate, consumers efficiently reduce the rate of pathogen population growth.

### Indirect pathogen flow

To better understand the spatial dynamics in the model, we define two quantities related to population-level movements: (I) an indirect pathogen flow and (II) an effective indirect pathogen flow. Contrary to direct pathogen flow, where pathogens disperse directly from one patch to another, the indirect pathogen flow describes pathogen movement due to dispersing infected hosts. To be precise, it is the amount of pathogen shedding of immigrating infected hosts. Let time points be $T=\{{t}_{1},\ldots ,{t}_{k-1},{t}_{k},{t}_{k+1}\ldots ,{t}_{n}\}$, where *t*_*k*_ is a time point with an index *k* (any continuous-time ODE solver discretizes a time domain). The strength of the *indirect pathogen flow* in the patch *j* at time *t*_*k*_ is:

7 $${\rm{ipf}}\,(j,{t}_{k})={d}_{{\rm{host}}}\lambda {I}_{i}({t}_{k}),$$

where *I*_*i*_(*t*_*k*_) denotes infected hosts at time *t*_*k*_ in patch *i*. This measure assumes that at the next time step an immigrant becomes a resident and its shedding is no more taken into account. However, if both patches have equal amount of emigrating infected individuals, the resulting change is zero. Therefore it is better to measure only the net gain:

8 $$u(i,{t}_{k})=\,{\rm{\max }}\,[0,{\rm{ipf}}(j,{t}_{k})-{\rm{ipf}}(i,{t}_{k})].$$

If the number of infected emigrants is larger than the number of infected immigrants, then *u*(*i*,*t*_*k*_) is zero. Now we can compute the *effective indirect pathogen flow* by approximating cumulative net gains during small time intervals that are formed by the sample points *T* of a ODE solver. By adding all these intervals together we obtain:

9 $${\mathscr{U}}(i,T)=\frac{1}{({t}_{n}-{t}_{1})}\sum _{k=2}^{n}\,(({t}_{k}-{t}_{k-1})\frac{u(i,{t}_{k})+u(i,{t}_{k-1})}{2}),$$

where the denominator of a fraction term in front of a sum is the length of a whole observation time interval. This function describes an effective average flow per time unit.

### Simulations

The differential equation system is simulated using the Matlab implementation of Dormand-Prince method (ODE45). In all experiments, a long enough transient (at least 2000 time units) is removed to ensure that the system has reached long-term behavior with a given parameter setting. We first visually tracked the lengths of transients using the overly long plots of given dynamics. In addition, we utilized also a doubling procedure, where we doubled the length of a run (and the transient) to ensure that the computed results stay same between two runs. This should confirm that the initial transient was selected to be long enough. The default parameter values were selected to ensure the persistence of the consumer and pathogen in the absence of a host.

## Results

### Single-patch dynamics and pathogen dispersal

**Figure 1:**
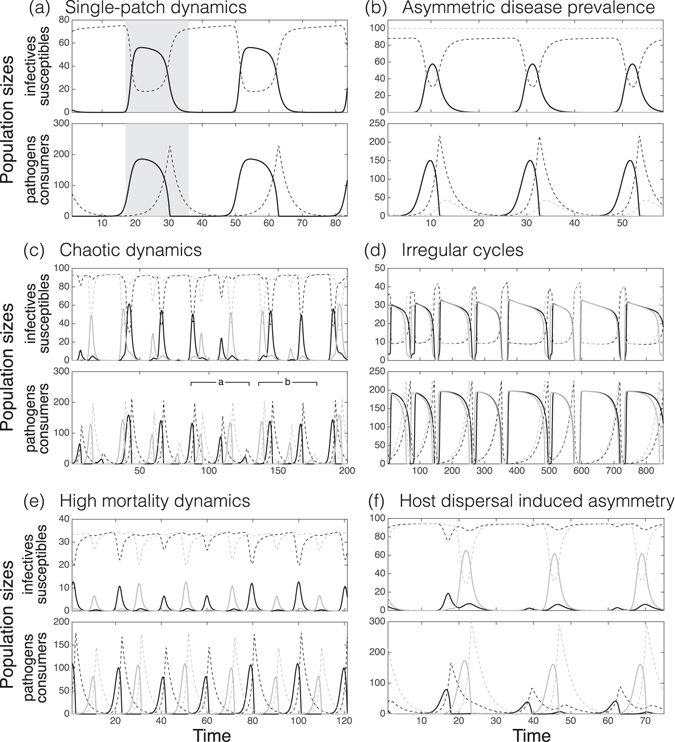
Six different representative classes of cyclic dynamics from model simulations. A line color indicates population’s habitat patch (a black or grey patch). In each picture, an upper panel shows the number of infected hosts (solid line) and susceptibles (dotted line) over time and a lower panel shows the number of pathogens (solid line) and consumers (dotted line) over time. (**a**) Single-patch dynamics (*a* = 0.55, *h* = 0.45, *κ* = 3, *λ* = 0.1), where shaded areas make easier to compare dynamics between infectives and pathogens. (**b**) Asymmetric disease prevalence case (via consumer dispersal) showing one infected and one healthy patch (*a* = 0.55, *h* = 0.25, *κ* = 3, *λ* = 1, *d*_con_ = 0.15). (**c**) Example of chaotic dynamics (*a* = 0.55, *h* = 0.25, *κ* = 3, *λ* = 0.1, *d*_host_ = 0.1, *ν* = 0.01), where intervals **a** and **b** show the effect of indirect pathogen flow (sequences of decreasing pathogen populations). (**d**) Example of irregular cycles (*a* = 0.55, *h* = 0.85, *κ* = 3, *λ* = 0.5, *d*_host_ = 0.1). (**e**) Example of dynamics under a high mortality rate (*a* = 0.55, *h* = 0.25, *κ* = 3, *λ* = 0.1, *d*_host_ = 0.1, *ν* = 0.15). (**f**) Asymmetric disease prevalence caused by host dispersal (*a* = 0.55, *h* = 0.25, *κ* = 3, *λ* = 1, *d*_host_ = 0.11). Notice that population sizes (y-axis) and time intervals (x-axis) vary between panels.

With the default parameters (Table 1), dynamics of an isolated, single-patch system is cyclic. The cyclic dynamics arises from a cyclic consumer-resource interaction, which is in turn mediated to pathogen-host interaction, resulting in infection cycles (Fig. 1a). The infectivity response controls how rapidly an epidemic builds up, and the consumer functional response mainly controls the length and decline of an epidemic.

The direct dispersal of pathogens between patches (e.g., via air, water, or unknown vectors) has a strong syncronizing effect on all populations. Even relatively small pathogen dispersal rates (*d*_path_ ≈ 0.01) are enough to synchronize the patches. This happens because the pathogen directly couples to the consumer as well as the host.

### Asymmetric disease prevalence under consumer dispersal

**Figure 2:**
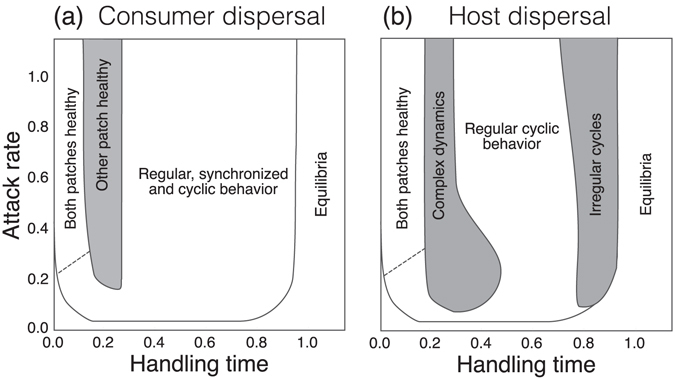
The effect of varying handling time and attack rate on host-pathogen dynamics. In general, cycle lengths increase when a handling time is longer. With a short handling time aggressive consumers make the system disease free, whereas almost all hosts are infected if the handling time is long. (**a**) Areas of different dynamics with the consumer dispersal rate of 0.25 (a large value selected to maximize the effect). (**b**) Areas of various dynamics with respect to the host dispersal rate of 0.1. While parameter values *κ* = 4 and *λ* = 0.5 were used to produce contrasting patterns, a large range of *κ* and *λ* values produces similar dynamical partitions. The dotted line describes a gradual shifting from one dynamics to another and solid borders show an abrubt change in a dynamical behavior. The drawn plots are based on simulation runs with a single representative initial value setting (*S*_1_ = 90, *I*_1_ = 0, *P*_1_ = 100, *Q*_1_ = 200, *S*_1_ = 10, *I*_1_ = 0, *P*_1_ = 100, *Q*_1_ = 10). Initial values have influence on shifts between different dynamics and therefore these plots give only approximate dynamical ranges. Hence, near the border area of two different dynamics, the system may end up either of stabilized dynamics depending on the initial balance between different populations.

Weak consumer dispersal synchronizes patches, due to its influence on the pathogen populations. However, the situation changes under stronger dispersal (e.g., *d*_con_ > 0.1). With highly aggressive consumers (handling time *h* < 0.1, coupled with high enough attack rate), the pathogen is unable to infect hosts, as the consumers keep pathogen densities below the infective threshold, imposed by the sigmoidal infectivity response (Fig. 2a). Unexpectedly, less aggressive consumers (0.1 ≤ *h* ≤ 0.27) generate a persistent asymmetry in disease prevalence, where only one of the patches is disease-free (Fig. 1b).

Synchronized, cyclic dynamics in both patches is achived with longer consumer handling times (0.3 < *h* < 0.9). Highly inefficient consumers (either high *h* or low *a*) are not able to suppress the pathogen, leading to the collapse of the cyclic dynamics. As a result, the system ends up in an equilibrium, where all three species coexist and a considerable proportion of hosts are infected (Fig. 2a).

We will next focus on the properties of asymmetric disease prevalence (Fig. 1b). Firstly, during outbreaks the total number of infected hosts is lower than in the case where both host populations are suffering from the disease. Secondly, the phenomenon is initial value dependent: When the dynamics of the patches are initially in the same phase (equal population sizes in both patches), asymmetry cannot take place. The larger the phase difference, the weaker dispersal is required for asymmetry to emerge. One patch becomes a consumer source that is able to send enough consumers to the other patch (a consumer sink) preventing the rise of epidemic outbreaks in that patch (thorough analysis is in SI, section A). Hence, even if the consumer peaks of the source patch are much higher (Fig. 1b) than the peaks of the sink patch, one determining difference is that the consumers are at the right moment present to prevent the emerging growth of pathogen population. The other difference is that the pathogen population is much smaller than in the source patch, and thereby a small amount of consumers can prevent the pathogen population to grow. On the other hand, there is no pathogen suppression in the source patch as the patch practically follows the single-patch dynamics without any immigrating consumers, Lastly, when carrying capacities of hosts between patches are dissimilar, a host-poor patch can still be a source, if it gets a strong initial advantage and consumer dispersal is strong enough to eradicate pathogen outbreaks in the host-abundant patch.

Sometimes a dynamical behavior that is present in a deterministic system, does not appear in the corresponding stochastic system (e.g, the required precise timing can disappear). Therefore we implemented a stochastic version, and it also shows asymmetry in disease prevalence (see SI, section D). The main difference is that sink and source periods alternate between the two patches due to stochasticity, which is expected as the timing of outbreaks varies and therefore consumers blocking the pathogen growth, may not be present at the right moment.

### Disease dynamics caused by indirect pathogen flow

**Figure 3:**
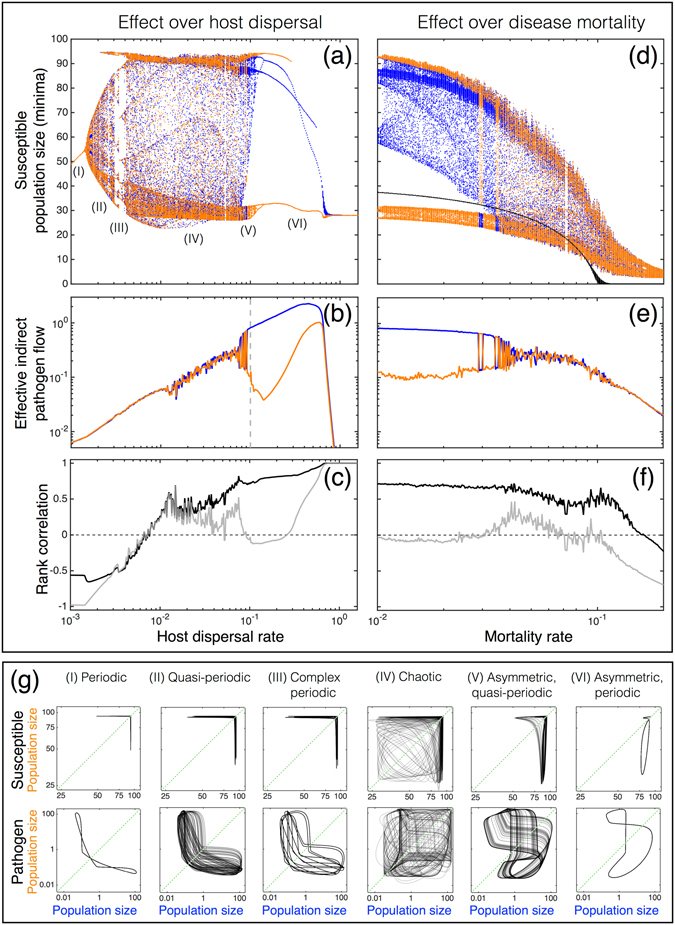
The bifurcation analysis of a chaotic case is presented over host dispersal and mortality rates. (**a**,**d**) Two bifurcation plots of susceptibles (*κ* = 3 and *λ* = 1). The colour indicates different patches (blue and orange). In (**d**), black points represent the bifurcation plot of a double-sized one-patch system (comparable host population size) showing extinction under a lower mortality rate than an asynchronous two-patch system. The next row (**b**,**e**) represents effective indirect pathogen flow caused by immigrating infected hosts. A dotted vertical grey line on (**b**) shows the host dispersal value (0.1), fixed in (**e**). Panels (**c**,**f**) show synchronization between the patches for susceptibles (black line) and for the pathogen populations (grey line). Panels (**a**–**f**) are computed using the same parameter values and therefore they show different aspects with respect to the same runs. (**g**) Attractors of the susceptible and the pathogen for different dynamical scenarios indicated in panel (**a**). Label colors match with patch colors. Along increasing host dispersal, the system starts from periodic dynamics (I), moves to quasi-periodic behavior (II) and after that follows chaotic dynamics (IV). As the pressure of indirect pathogen flow further increases the system becomes first asymmetric, quasi-periodic (V) and then asymmetric, periodic (VI). Complex periodic dynamics (III) take place in bifurcation diagrams during phase-locking periods (the behavior of a system becomes occasionally simpler).

**Figure 4:**
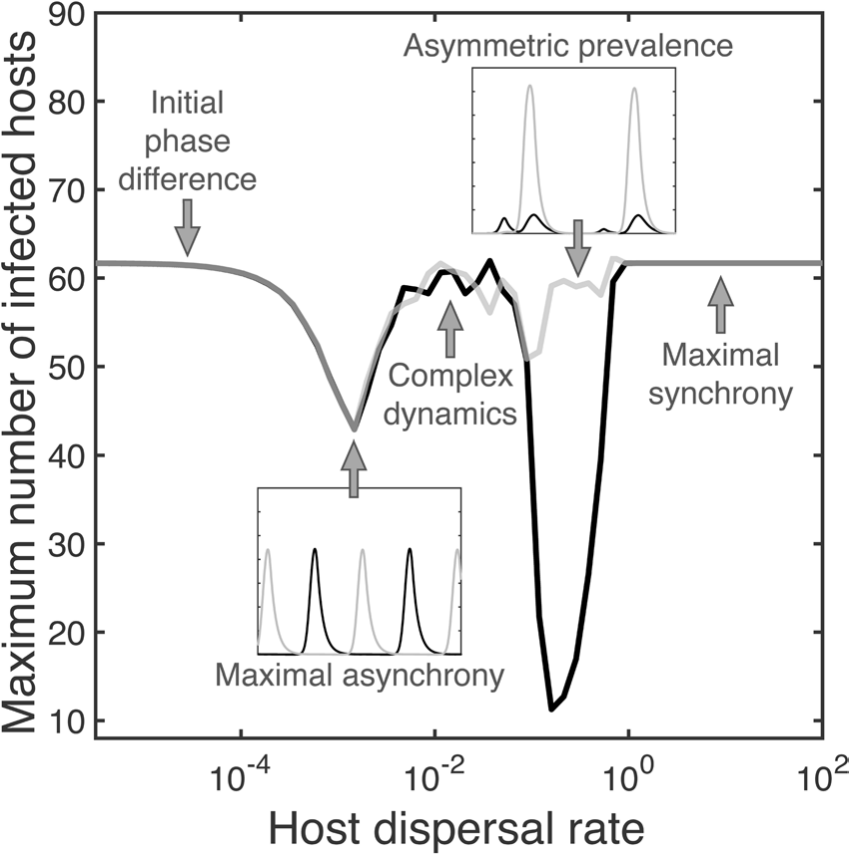
Host dispersal influences the sizes of epidemic outbreaks. Maximum outbreak sizes of two patches are denoted with grey and black curves (averaged over 50 randomly initialized runs, *a* = 0.55, *h* = 0.25, *κ* = 3, *λ* = 1, *ν* = 0.01). With very small rates, the two patches are practically independent in their dynamics. The minor movement of infected hosts pushes patches to maximal asynchrony (a lower small panel shows the dynamics of infected host populations) due to better consumer persistance. After the host dispersal range of symmetric complex dynamics, even stronger indirect pathogen flow causes asymmetry in disease prevalence (an upper small panel shows the part of a single run). Finally, the patches become fully synchronised with extremely strong dispersal.

Assuming that infected hosts continue to disperse, their movement creates an indirect flow of pathogens between patches. This reduces the net loss rate of the consumer population and therefore reduces the growth of the pathogen population. This, in turn, acts to dampen the following epidemic outbreak in a patch (see Fig. 1c). When consumer handling time is relatively short (Fig. 2b), outbreak sizes and their intervals vary irregularly–showing chaotic dynamics (Figs 1c and 3), but the system is still interpretable within short time intervals (SI, section B). Stronger shedding (*λ*) or increased host dispersal rate (*d*_host_) acts to increase the indirect pathogen flow. This leads to asymmetric disease prevalence (Figs 1f and 4), due to sustained directional net flow of pathogens from one patch to the other (Fig. 3b). As a result, the consumer population remains at sufficiently high density at all times in one patch.

Increased disease mortality among the hosts weakens the indirect pathogen flow, as infected hosts die more rapidly (Fig. 3e). In addition, if the mortality rate (*ν*) is sufficiently high, between-patch dynamics become asynchronous (Figs 1e and 3f). In turn, this promotes host metapopulation persistance, as compared to more synchronous dynamics (Fig. 3d, SI, section C). When handling time is long (Fig. 2b), associated with long-lasting disease outbreaks, indirect pathogen flow generates irregularities on the durations of epidemic outbreaks (Fig. 1d). Here the dynamics can be divided into two phases. In an expansion phase, indirect pathogen flow extends the difference between cycle duration between patches. In a contraction phase both cycles become much shorter than without indirect pathogen flow (Fig. S3; see SI, section B for additional information).

We use the term *complex dynamics* to consists of all dynamics from complex periodic deterministic dynamics to chaotic dynamics. The division between complex dynamics and irregular cycles is made, because indirect pathogen flow acts different way in these two categories (see SI, section B for additional information).

### Density-dependent host dispersal: susceptibles avoid infected hosts

The asymmetrical disease prevalence can also be achieved by modifying dispersal assumptions. Here we consider a density-depended dispersal scenario, where infected hosts are not able to disperse and susceptibles try to avoid them by moving to the other patch (without knowing the situation there). The movement of susceptibles is increased in response to the number of infected hosts *I*_*i*_ in a patch *i* as *m*exp(*αI*_*i*_) (a related idea in Abrams *et al*.^31^). The parameter *m* is a basal dispersal rate and *α* determines the steepness of the exponential curve. When the curve is very steep, susceptibles have a strong avoidance towards infected hosts. This behavior has three consequences: (I) infected and susceptible hosts occupy mostly different patches, (II) epidemics stay small as there are no susceptible hosts present, and (III) the total amount of hosts decreases as one of the two patches is almost abandoned. On the other hand, if we make the basal dispersal rate and the steepness of the exponential function nearly independent of each other and increase the basal dispersal rate, synchronization between the patches increases and the avoidance effect thereby will become weaker. Thus, the resulting dynamics is a combination of basal random movement and avoidance behaviour.

## Discussion

We found that in a spatial host-pathogen-consumer system, consumer and host dispersals can lead to an emergent asymmetry in disease prevalence between patches, as well as more complex local population dynamics. While spatial asymmetry due to consumer dispersal is easy to understand (a spill-over of consumers from one patch maintains the other patch disease-free), it is surprising that the same effect can also emerge under host dispersal (Figs 1f and 4). This happens as strong indirect pathogen flow can maintain a consumer population at a sufficiently high density to suppress pathogen population growth in the outside-host environment. Unlike with consumer dispersal, the healthier patch is not completely disease-free, due to immigrating infected hosts and a relatively slow consumer population response to pathogen population growth. Counterintuitively, this leads to a situation where immigrating infected hosts decrease disease prevalence in a focal patch instead of increasing it (complex dynamics turns into asymmetric disease prevalence case in Fig. 4). At the same time, the host population size stays close to the carrying capacity of the patch, which provides additional evidence that the effect is not caused by somehow increased mortality among infected hosts. These effects persists, even if we add 10% dispersal mortality among consumers or hosts, as long as we compensate the loss of migrants by increasing growth and dispersal rates.

While spatial heterogeneity in population sizes is usually attributed to differences in environmental conditions and/or overall patch quality, which can in turn promote source-sink dynamics or mass effects^32^, our results indicate that consistent spatial asymmetry can also arise in a fully homogeneous system. In this case, asymmetry is generated by the interplay between interspecific interactions and dispersal. Existing theory on competition assumes that if the competitive environment is homogeneous, coexistence is only possible if there is sufficient intraspecific aggregation in species spatial distributions^33^. Such aggregation—which is commonly observed in natural systems^34^—has not been expected to arise without some ecological differentiation between species^35^, or heterogeneity in environmental conditions^36^. Recently, emergent asymmetry in population densities—due to spatial aggregation—in a homogeneous system has been shown to arise under reproductive interference^37^. It is clear that adaptive behaviour, such as movement, can easily produce out-of-phase dynamics between patches^38^, as also shown here, or sustained asymmetric spatial distributions in heterogeneous space^39^. However, our analysis demonstrates that asymmetric disease prevalence, and thus host population size, between identical habitat patches can arise either via a mass effect generated by consumer dispersal, or by indirect pathogen flow due to dispersing infected hosts, associated with increased consumer growth rate in the recipient patch.

Empirical studies are supporting consumer-pathogen dynamics showing density dependence^40^, prey preference (in case of E. coli K-12)^13^, and cyclic behavior between protozoa and prey^41^, although evolution may affect the latter within some time scale^42^. Pathogen reservoirs can be found from environment^1^ and protozoan predators are able to regulate pathogen populations^9^. Host actions^43^ and dispersal^28^ spread EOPs and EOP diseases in a landscape. The direct empirical evidence between a strong pathogen flow and decreased infectivity in host populations is missing with respect to our system. However, it has been shown that in highly connected areas (strong pathogen flow), a fungal plant pathogen experiences higher extinction rates than expected due to the higher level of disease resistance^44^. In our system, the strong indirect pathogen flow increases the number of consumers (instead of host resistance), which on its behalf keeps the hosts healthy.

Asymmetric disease prevalence due to consumer dispersal demonstrates a possible biological control mechanism: a small amount of consumers can introduced at a precise moment to prevent the strong growth of the pathogen population taking place a moment later. Other two options, a constant consumer flow and the introduction of consumers after an outbreak, are less effective. The first one can lead to pathogen immunity against consumers, due to the constant presence of the consumers, and in the latter case, there are already many infected hosts present and a relative large amount of introduced consumers is needed for killing the ongoing epidemics. There have been experiments for using predatory species for biological control^41, 45, 46^ and Merikanto suggested that fisheries could fight against columnaris disease using an artificially created protozoa inflow^8^. Here, we do not model any evolutionary changes in the consumer and pathogen species. However, in reality pathogens are taking many countermeasures (formed via evolution) to survive from protozoan predation, for example using toxics, surviving in hostile intracellular environment, or by forming biofilm^47, 48^. The effectiveness of biofilm as protection against predation can, however, be context specific, as some protozoa can successfully feed biofilms^45^. In this study, we also expected a constant virulence throughout time, but in reality pathogen virulence is under evolutionary change as well^49, 50^.

Pathogen consumers in the outside-host environment can also play an essential role in the stability of local dynamics. In our two-patch metacommunity, both communities contain a food chain of three trophic levels (rather similar to the food chain of hyperparasitism). The bottom level species (host) forms a stable, rich food source for the intermediate consumer, i.e. the pathogen. This might be the reason why we see only stabilizing synchronous effect with pathogen dispersal, although a dispersing intermediate predator species can be destabilizing as well^51^. In general, the synchronizing effect of an intermediate level is the strongest as it couples the top and bottom levels^52^. This is also the case in our model, where pathogen dispersal has the strongest synchronizing effect. Here, dispersal at the bottom level is actually hierarchical, as it also causes the movement of the intermediate level species (pathogen), unless host dispersal is restricted to healthy individuals. This sort of hierarchical dispersal can be destabilizing^53^. Finally, the top level is occupied by pathogen-eating consumers. Consumer dispersal—coupled with practically non-diminishing bottom level resources—collapses the system essentially into a di-trophic food chain, where the pathogen population of one patch may become extinct due to consumer dispersal. Dispersal with respect to different tropic levels can have dissimilar effects on the dynamical behavior of a system, as discussed also in Koelle and Vandermeer^54^.

Any seasonal effects or other stochastic variation, which were not included in the model, can potentially have a huge impact on the dynamics. From one season to the other, growth and mortality rates of different species, as well as dispersal rates, can change in response to changes in environmental conditions. The effects of seasonal and stochastic environmental variation have been studied for example in Anttila *et al*.^19, 20^, where environmental fluctuations were targeted to have effects on virulence and microbial population sizes. Stronger variation was shown to produce more severe epidemic outbreaks in both cases. In our system, persisting consumer populations can reduce the severity of such outbreaks. The influence of environmental fluctuations on populations in metacommunities can further depend on species dispersal capacity, relative to other members of the community^55^. In addition, how different populations are affected by the environment can also be important^19, 55, 56^. If we expect distinct environmental fluctuations in each habitat patch, dispersal acts like an averaging force on patch demography. This, however, could mean that an epidemic can spread also to other patch due to host or pathogen dispersal, or dispersing consumers are able to supress an arising epidemic rapidly. Hence, demographic averaging does not necessary mean a waning epidemic, but the current state of the system, as well as the dispersal type have a strong impact in each particular case.

While we have assumed a type II saturating functional response for the consumer, the shape of the response is relevant for the presented results. We also tested the sigmoidal response of type III, which is shown to give more stable dynamics than the type II^57^. The areas of complex dynamics and irregular cycles tend to change their dynamic behavior (Fig. S6), shrink, move in the parameter space and even disappear, when tuning the functional response towards the type III. This in turn indicates that the particular shape of the response causes the most observed irregularities. The stabilization happens, because the response of type III is removing the contributions of the smallest pathogen populations. We confirmed this, by shifting the response of type II to ignore the smallest populations and achieved the same effect. A linear functional response (type I) is incompatible with the presented dynamical ranges as it lacks handling time (*h*) and attack rate (*a*) parameters. However with the type I, the system shows either equilibrium behavior or regular cyclic dynamics.

Our model bares some similarity with that of Moore *et al*.^18^, who analysed the effect of predation on a vector-borne disease. The current model is not directly applicable to vector-born diseases in general (e.g., due to the assumption of host-independent pathogen growth), but could be easily modified to study the role of dispersal in the dynamics of vector-born diseases. While this is not within the scope of the present paper, it is an interesting topic for future research.

Our present research reveals that consumer and host dispersal can lead to asymmetric disease prevalence between patches in a homogenous system. Strikingly, morbidity of a focal patch can decrease under increasing host dispersal, although the immigration of infected host becomes stronger at the same time. This happens due to indirect pathogen flow, which arises when pathogens use dispersing hosts as vectors. This flow is also causing complex dynamical effects: outbreak sizes and intervals or their durations become irregular. All these unexpected dynamics will take place only when consumers interact with a host-pathogen system, which emphasizes that (I) a community structure in a free-living stage of the pathogen matters and therefore (II) the constituent species in a community cannot be always safely omitted, as they can have radical effects on disease dynamics^16^. Our findings add to the importance of metacommunity interactions in spatial disease epidemics. The link between community ecology and disease dynamics has been recognized as important^58, 59^, but we stress that species interactions do not occur only between larger, more complex life forms, but also the communities of microbial life forms can have large effects on disease dynamics.

## Electronic supplementary material

Supplementary Information
