# Supplementary material for: Spatial disease dynamics of free-living pathogens under pathogen predation
Source: Sci Rep. 2017 Aug 10;7:7729. doi: 10.1038/s41598-017-07983-2 (PMC5552698; doi:10.1038/s41598-017-07983-2)
Supplement: Supplementary file 1 — Supplementary Information [file 41598_2017_7983_MOESM1_ESM.pdf]

SUPPLEMENTARY INFORMATION
Spatial disease dynamics of free-living pathogens
under pathogen predation

Tommi Mononen and Lasse Ruokolainen

A. Further analysis of asymmetric disease prevalence under consumer dispersal

We will analyze the case with asymmetric disease prevalence more carefully. Firstly, during outbreaks the total number of infected hosts is lower than in the case where both host populations are suffering from the disease. To measure the severity of epidemics, with given initial values, we use the measure:

$$\text{minmax infectives} = \min_{d_{\text{con}}} \max_{\{t_1,\ldots,t_n\}} (I_i(t_k) + I_j(t_k)), \quad (1)$$

which counts the largest number of infected individuals over time, using the minimizing consumer dispersal rate (I_i is the number of infected hosts in patch i, d_{con} is a consumer dispersal rate and t_k is the kth time point). The idea is to investigate, how consumer dispersal rates and phase differences are coupled with each other. To compute an underlying base cycle (single-patch dynamics with given parameters), we switched dispersal off. Using this base cycle, we adjusted an initial phase difference between the patches (e.g. the second patch begins its pathogen growth 1/2 cycle later than the first patch) and turned dispersal on for an actual simulation run.

When the populations in two patches are initially adjusted to the same cycle phase (no phase difference), they stay in synchrony and no asymmetry emerges (unless we perturb slightly one patch). Generally, as a phase difference is quite large, the number of infected hosts is lower due to asymmetry and this asymmetric effect takes place already with a low consumer dispersal rate (Fig. S1a,c). Increasing consumer dispersal rate increases the number of infected hosts (Fig. S1a). With larger dispersal rates, many consumers end up into the patch low in pathogens, resulting in lower overall pathogen consumption.

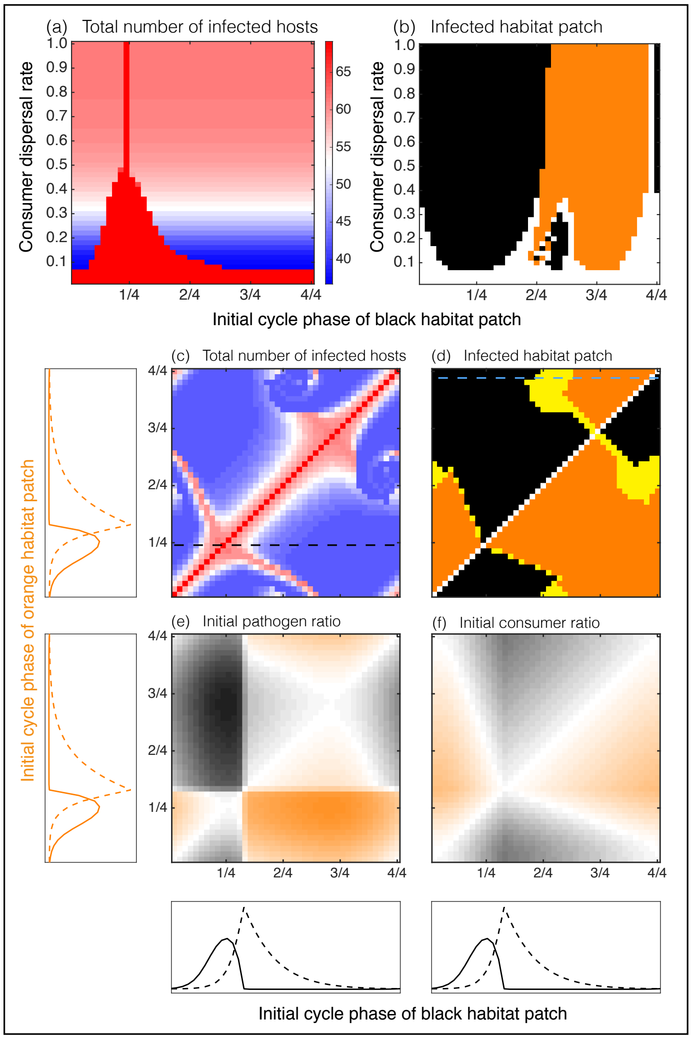

Figure S1: The total number of infected hosts (the max peak, which equals the worst case) in the system (over a long time period) is initial value dependent in the case of asymmetric disease prevalence ($a = 0.55$, $h = 0.25$, $\kappa = 3$, $\lambda = 1$). (*a*) Effect of a consumer dispersal rate with respect to the phase difference between two patches. Initial values of one patch are fixed (marked with a black dotted line in (*c*)). High infectivity areas with small dispersal values indicate the areas, where dispersal is not strong enough to push disease prevalence asymmetric (a saturated red color in (*a*). In general, higher dispersal rate values show a higher number of infected hosts, because the consumers are dispersing strongly from a pathogen-rich patch to a patch without any pathogens. (*c*), Plot shows the smallest possible number of infected hosts for given initial values (shown in marginals: solid is the pathogen, dotted is the consumer) with disease minimizing consumer dispersal rates (the minimization goes over y-axis of (*a*)). (*d*) shows areas, where the orange patch is under disease (orange color) and the black patch is under disease (black color). In the white diagonal both patches are synchronized and their hosts get infected. Yellow areas indicate the locations, where the healthiness of a patch is dispersal rate sensitive. (*b*) Example of this kind of behavior can be seen around 2/4 cycle shift. With small dispersal rates the black patch is suffering from disease, but with higher rates the orange patch becomes infected instead. A white color means an almost symmetric dynamic behavior. The slice position is marked with a dotted blue line to (*d*). (*e*) and (*f*) explain differences in the numbers of infected hosts by showing the logarithms of initial value ratios of pathogens and consumers. Black and orange shades indicate magnitude differences of the initial values. A saturated orange for example, denotes that the corresponding initial value of the orange patch is much greater than an initial value in the black patch. A white color means that both populations are equal-sized.

In general, the patch with initially higher pathogen density remains infected (compare Figs. S1d and S1e). Consumers emigrating from this patch will eradicate the pathogen population in the other patch, which will therefore become disease-free. However, if pathogen densities are initially at the same level, or the first patch is low in pathogens and high in consumers and the second patch has the opposite composition, then the situation becomes more complex (Fig. S1c–f). In the latter case, an infected patch may become healthy and a healthy patch infected, when a consumer dispersal rate is increased (Fig. S1b). This change happens, because with the high dispersal rate the initial consumer population sizes become irrelevant (in compared to the pathogen population sizes) as consumer populations mix quickly.

Interestingly, the asymmetric disease prevalence is not very sensitive to differences in host carrying capacities; a patch with a smaller host population can still eradicate the pathogen from a patch with a larger host population. Hence, an initial phase difference is enough for a consumer population to kill all pathogens from the larger patch. However, the consumer dispersal rate has to be greater for controlling larger infected host and pathogen populations (especially with initial values that do not favor strongly dominance of the smaller patch). Stronger shedding of the larger patch turn the tables easily on its favor.

Whether asymmetric disease prevalence can arise depends crucially on the shape of the infectivity function. When $\kappa = 1$ the asymmetry does not emerge; the infectivity function is only saturating, not sigmoidal (Fig. S5a). In this case there is no infection threshold and thus pathogens are able to infect hosts even at low densities. As a result, a small consumer population is not able to control the growth of pathogens, supported by shedding from infected hosts, which leads to synchrony between the patches.

B. Chaocity of the system and further analysis of host dispersal

A system is said to be chaotic, if a very small perturbation to an initial state leads a deterministic system to a state that cannot be predicted given the system, the unperturbed initial state and time elapsed. Moreover, the system has to be dissipative, which essentially means that trajectories are bounded in space. Therefore the system always returns close to states it has visited before (we have an attractor). Chaotic behavior can be tested using Lyapunov exponents. Approximate Lyapunov exponents (Matlab® script: *Calculation Lyapunov Exponents for ODE*, MathWorks® repository, Vasiliy Govorukhin, 2004) computed for our system confirm chaotic behavior that is visible also in bifurcation plots (Fig. 3a,d) and attractors (Fig. 3g, especially the attractor IV). According to Lyapunov exponents, the dynamics of the ODE system is weakly chaotic (Fig. S2) (Dingwell, 2006), and becomes unpredictable after tens of outbreak cycles, but remains interpretable within short time intervals (Wolf et al., 1985).

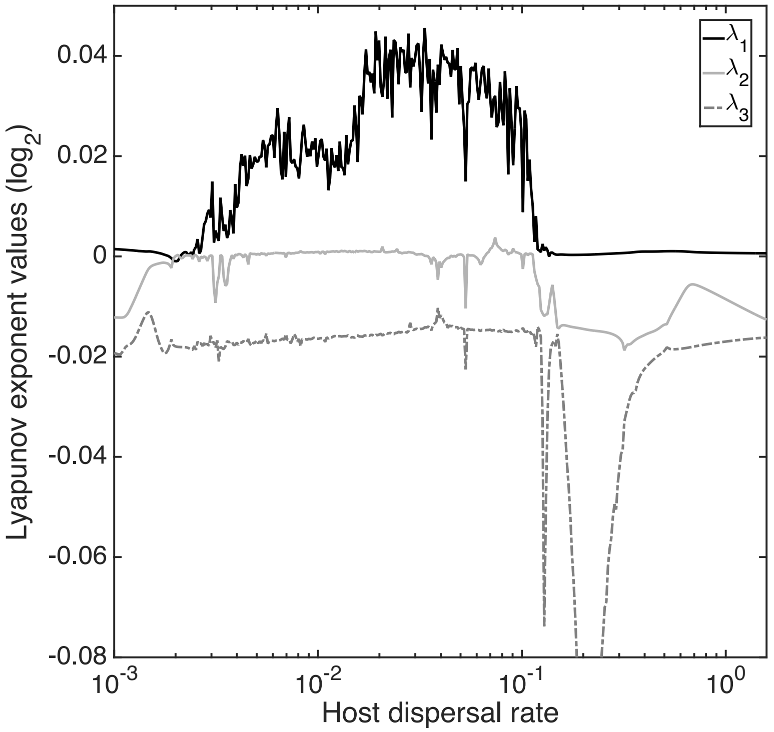

Figure S2: Three largest Lyapunov exponents (of eight) are estimated for our model. An ODE system is chaotic when the largest Lyapunov exponent λ_1 is positive and the system is dissipative (one of the exponents is negative, here λ_3). One of the exponents has to have zero value according to a theory (here either exponent λ_1 or λ_2 take this role). The largest Lyapunov exponent indicates the magnitude of chaocity of the system, meaning how rapidly the system becomes completely unpredictable given a slightly perturbed initial state. In this case, the chaotic effect is quite weak but still present. The model parameter values are the same that was used in the computation of Fig. 3a–c (κ=3, λ=1, a=0.55, h=0.25). Notice that the exponents describe the whole system, not just the behavior of a time sequence for susceptibles.

Our basic assumption here is that infected hosts disperse with the same rate as susceptibles. The movement of infected hosts creates indirect pathogen flow, causing two novel dynamical effects: complex dynamics (consists of all dynamics from complex periodic deterministic dynamics to chaotic dynamics) and irregular durations of disease cycles (Fig. 1c,d). We divide these two dynamics into different categories by naming them differently as indirect pathogen flow is taking action dissimilarly in them. In the following, we will explain the underlying mechanisms of these phenomena.

Short handling time (Fig. 2b: Complex dynamics)

When the consumer handling time is relatively short, the dynamics become chaotic (according to Lyapunov exponents) with small host dispersal rates (Fig. 2b). Epidemic outbreaks do not appear within regular intervals and the sizes of these outbreaks can vary considerably, as seen in Fig. 1c. The main factor causing the complex dynamics is the indirect pathogen flow (pathogens spread with immigrating infected hosts) that feeds an existing consumer population; consumer decay is slower than without the flow. This in turn, restricts the size of the next pathogen population growth phase in a patch, as the consumer population can recover faster than without the flow. When the same phenomenon happens several times in a row, a sequence of shrinking outbreaks results (areas *a* and *b* in Fig. 1c). This is due to the increasing baseline population size of the consumer population with every new outbreak. At the same time, the interval between disease outbreaks shrink, as each new peak of the pathogen population can start sooner as consumer population maxima become smaller. The different rhythms between the patches (caused by the shrinking intervals) make the effective indirect flow change its direction. Hence, over time the dynamics is driven by the constant direction changes of the effective indirect pathogen flow.

Increasing host dispersal makes indirect pathogen flow larger between two patches and this pushes the system first from regular to chaotic dynamics and after that to asymmetric disease prevalence dynamics (Fig. 3a,g). In this case, the asymmetry arises as pathogen inflow of one patch is maintaining a consumer population in the other patch (Fig. 3b). However, while disease prevalence is asymmetric, the patch with net inflow is not completely disease free, due to immigrating infected hosts (Fig. 4). With extremely high dispersal rates the pathogen populations become synchronized, as two host populations are effectively united by strong movement (Fig. 3c).

Host dispersal and disease mortality are opposite forces with respect to the indirect pathogen flow. Host dispersal increases indirect pathogen flow by increasing the movement of infected hosts. Disease mortality, on the other hand, reduces this flow by killing infected hosts and thereby it reduces pathogen shedding directly, and also by decreasing host population sizes. Therefore by increasing mortality, the effective indirect pathogen flow decreases and dynamical changes take place in the reversed order, as compared with increasing host dispersal (Fig. 3d,e).

Long handling time (Fig. 2b: Irregular cycles)

When consumers are inefficient (a long handling time, Fig. 2b), a consumer population responses slowly to the growth of a pathogen population. The chaotic dynamics transforms into the dynamics of varying length cycles (Fig. 1d), and the dynamics can be divided into expansion and contraction phases.

The expansion phase starts when the cycles of two patches end up in near-synchrony (see e.g., Fig. 1d). The life-time of the cycle starting pathogen population is getting shorter and the life-time of the following pathogen population longer, which increases spatial asynchrony (Fig. S3). The cycle starting patch gain additional shedding (via dispersing infectives) from the following patch, which in turn nourishes its consumer population. As the infected hosts immigrate to the starting patch, the pathogen population of the following patch survives longer due to a bit slower growing consumer population.

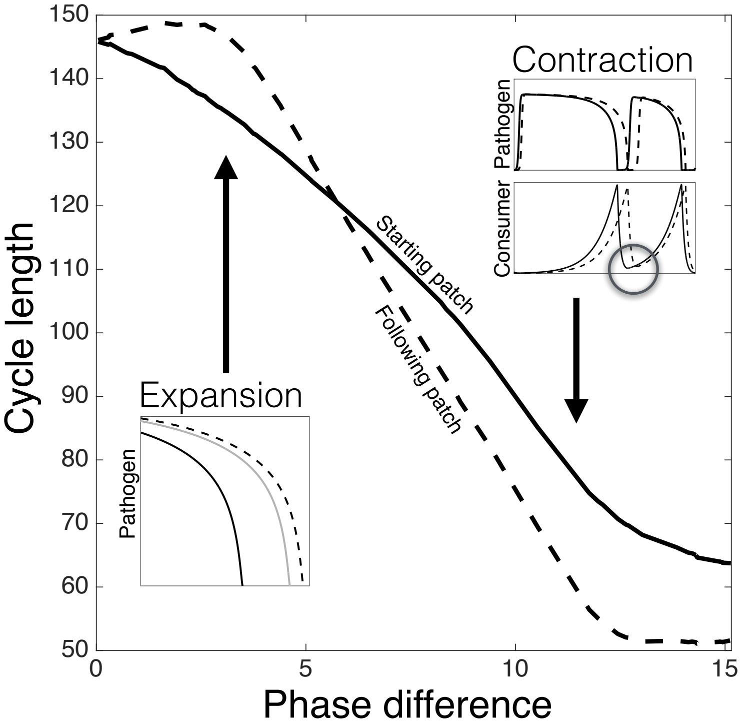

Figure S3: A graphical presentation that explains dynamics of varying cycle durations. The main plot shows that with smaller phase differences pathogen populations tend to asynchronize (expansion) and with larger phase differences to return in synchrony (contraction). A phase difference is defined using the distance between a close by consumer population peaks. An expansion panel shows the effect of asymmetrical indirect pathogen flow pushing populations apart from each other (line types match throughout the whole figure). A grey line shows the decline of pathogens without indirect pathogen flow. A contraction panel shows that large asynchrony stops the decline of consumer populations (the area inside a circle). This happens due to indirect pathogen flow, which keeps consumers alive.

The contraction phase starts when the phase difference is great enough (Fig. S3). The main reason is that the cycle starting patch begins the pathogen growth of next cycle before the following patch has completed the decline of its consumer population. The asynchrony stops the declines of both consumer populations reducing the length of next cycles in both patches. The next cycle of the following patch end up to be shorter, because the consumer decline is stopped in the earlier phase, leading the system again back to more synchronous state. The patches can swap their order (which patch starts a cycle first) due to an over-large contraction event, but this requires that the infectivity function is steep enough ($\kappa \geq 3$).

C. Asyncrony increases persistance

Large-scale spatial synchrony can compromise metapopulation viability due to increased risk of global extinction (Heino et al., 1997). However as Abbott (2011) showed, in some systems it is also possible that stability and synchrony are positively correlated with each other. In our two patch system, synchrony is positively associated with the extinction risk. A synchronized system (double-sized single patch) goes extinct with a smaller mortality rate than an asynchronous system (Fig. 3d). In the synchronous system, epidemics become larger (compare for example asynchronous and synchronous states in Fig. 4) causing the higher number of dead hosts, but after an epidemic the disease disappears for a while. In the asynchronous system one host population recovers, while the other is suffering from infection, such that the disease is always present at landscape level (Fig. 1e).

An increasing mortality rate seems to push disease outbreaks into asynchrony (Fig. 3f) unless indirect pathogen flow is too strong, causing strong coupling between the patches. We hypothesize that the phase difference between patches increases, because less hosts die under smaller asynchronous epidemics and more hosts die under larger mutually occurring epidemics. However, this drifting hypothesis cannot be validated based on our population level model; a formal validation would require an individual based system.

D. Stochastic version of the model

Sometimes stochasticity removes effects that are present in a deterministic differential equation system. Here we show that asymmetric disease prevalence survives in a stochastic system. We utilize classical Gillespie's Stochastic Simulation Algorithm (SSA) that was designed to simulate chemical reactions (Gillespie, 1977), but which is heavily used also in ecological scenarios. We used a Matlab algorithm found from MathWorks repository (Nezar Abdennur, 2012/version 1.3).

A set of ODEs can be transferred into propensity functions and state change vectors (e.g., the propensity function of infected host death in population 1 is νI_1 and when this event takes place, the population of infectives is reduced by one, which is described with a state change

vector) (Pineda-Krch, 1997). In addition, we encode our assumption of consumer and pathogen population persistances by restricting minimum sizes of these populations to 1. Hence, we expect that a population does not go extinct entirely, but always some individuals survive to ensure new growth.

First, we can compare the stochastic version of a cyclic disease dynamics in a single patch (Fig. S4a) to deterministic one (Fig. 1a) and see that they qualitatively match with each other as expected (using the same parameter values). Secondly, as we compare asymmetric disease prevalence under consumer dispersal, we notice that asymmetry still exists (Fig. S4b), but only for a relative short time until the consumer flow changes its direction due to random effects. Thereby the patch with infected hosts becomes healthy and the previous healthy patch start to suffer from disease outbreaks. Lastly, as asymmetry induced by host dispersal is weaker, stochasticity swaps the roles of the patches even more easily, but the underlying effect stays qualitatively the same.

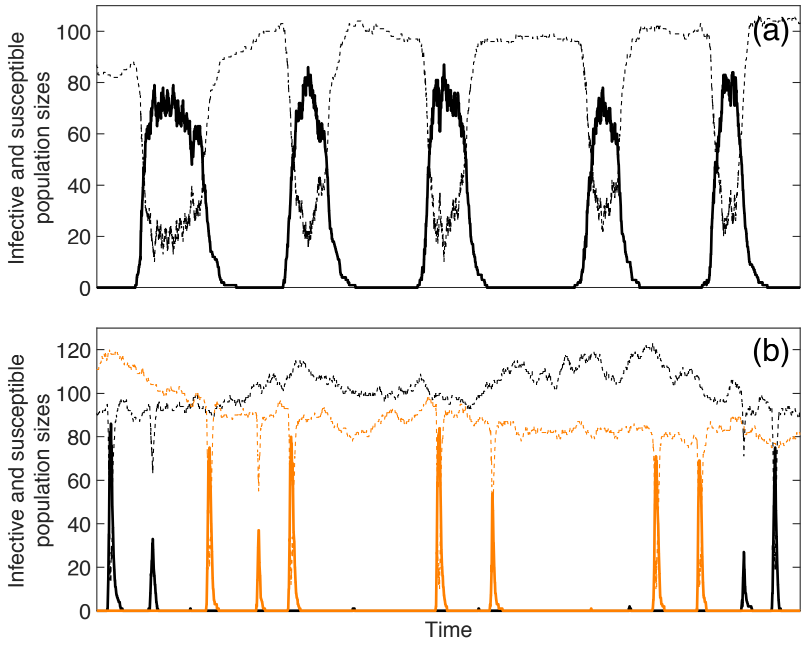

Figure S4: (a) Stochastic single patch disease dynamics, where a solid line shows the number of infectives and a dotted line is the number of susceptibles ($a = 0.55$, $h = 0.45$, $\kappa = 3$, $\lambda = 0.1$). (b) Asymmetric disease prevalence in the stochastic case induced by consumer dispersal (the effective pathogen flow is tuned to be on a bit higher level than in the deterministic case). Black and orange colors indicate two patches and the line type indicate infectives (solid) and susceptibles (dotted).

E. Additional figures

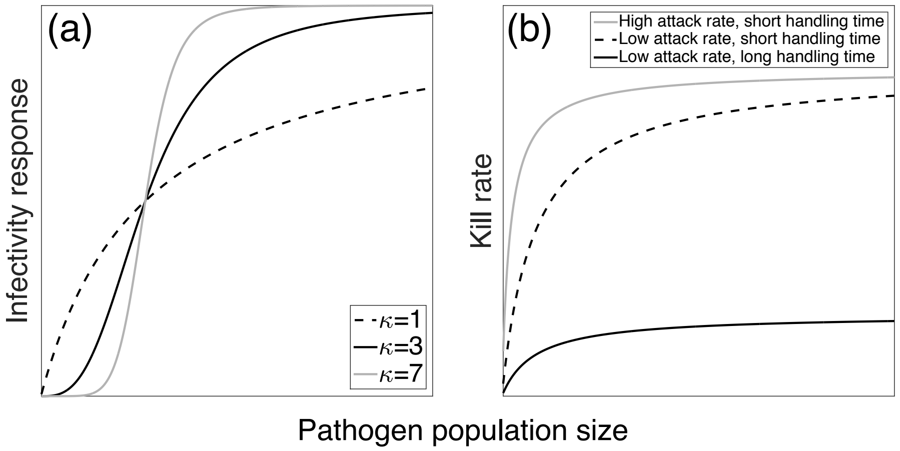

Figure S5: (*a*) Sigmoidal infectivity function with different values of κ. (*b*) Shape of Holling's functional response (type II) with three different combinations of handling time and attack rate. Values increase to the right and up.

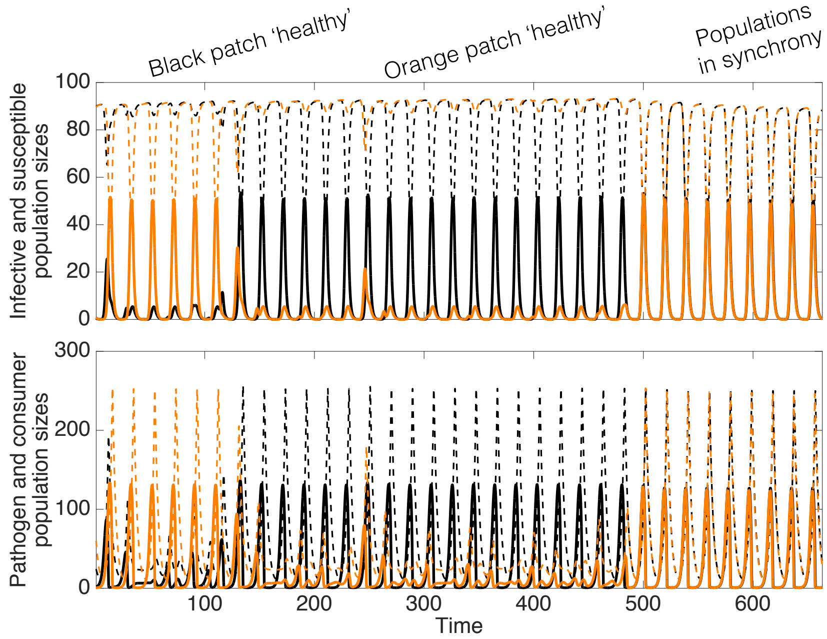

Figure S6: Chaotic dynamics using Holling's functional response tuned towards to type III ($q = 1.5$). A single simulation is showing both, synchronization and asymmetric states. More complex, chaotic dynamics can take place during transitions between these states (not shown in the figure). When $q > 1$, the area of the complex dynamics moves towards longer handling times and lower attack rates in a parameter space (a, h). The presented dynamics can be found with a parameter setting $a = 0.11$ and $h = 0.45$ under host dispersal. This particular area shows only regular dynamics with Holling's functional response of type II.

References

Abbott, K. C. 2011. A dispersal-induced paradox: synchrony and stability in stochastic metapopulations. Ecology Letters 14:1158-–1169.

Dingwell J. B. 2006. Lyapunov exponents. Pages 1-11 *in* Wiley Encyclopedia of Biomedical Engineering. Akay M. ed. John Wiley & Sons.

Gillespie, D. T. 1977. Exact Stochastic Simulation of Coupled Chemical Reactions. The Journal of Physical Chemistry 81(25):2340-–2361.

Heino, M., Kaitala, V., Ranta, E., and Lindström, J. 1997. Synchronous dynamics and rates of extinction in spatially structured populations. Proceedings of the Royal Society B: Biological Sciences 264(1381):481—486.

Pineda-Krch, M. 2008. GillespieSSA: Implementing the Stochastic Simulation Algorithm in R. Journal of Statistical Software 15(12):1-18.

Wolf, A., Swift, J. B., Swinney H. L. and Vastano, J. A. 1985. Determining Lyapunov exponents from a time series. Physica 16D:285–317.
